# miR-559 Inhibits Proliferation, Autophagy, and Angiogenesis of Hepatocellular Carcinoma Cells by Targeting PARD3

**DOI:** 10.1155/2022/3121492

**Published:** 2022-09-05

**Authors:** Chunjing Wang, Chengcheng Li, Rui Hao

**Affiliations:** ^1^Department of Gastroenterology, Affiliated Hospital of Beihua University, Jilin, Jilin Province 132000, China; ^2^Department of Graduate Student, Affiliated Hospital of Beihua University, Jilin, Jilin Province 132000, China; ^3^Department of Infectious Liver Disease, Affiliated Hospital of Beihua University, Jilin, Jilin Province 132000, China

## Abstract

Hepatocellular carcinoma (HCC) is one of the most common cancers in the world and has a high mortality rate. Although prevention and treatment of HCC has improved, it still faces poor prognosis and high mortality. miRNAs play a critical role in the tumorigenesis of HCC, but the underlying mechanism has not been well investigated. Here, the functions and interaction between miR-559 and PARD3 were investigated in HCC cells. Increased PARD3 and decreased miR-559 expression were observed in HCC cells compared with those in normal liver cells, especially in Huh-7 cells. Studies further demonstrated that PARD3 silencing or miR-559 overexpression impaired the proliferation, autophagy, and angiogenesis in Huh-7 cells. Mechanistically, PARD3 represents a target of miR-559. Furthermore, investigations revealed that miR-559 inhibition induced the expression of PARD3, thereby enhancing cell proliferation, autophagy, and angiogenesis in Huh-7 cells. These results reveal the interaction between miR-559 and PARD3 in HCC cells and provide new insights into their potential targets as therapeutic treatment against HCC.

## 1. Introduction

Hepatocellular carcinoma (HCC) is one of the most common malignancies in the world [1]. Although increasing studies are focusing on HCC, the underlying mechanisms about its tumorigenesis and development are still unclear. Since the proliferation and metastasis of tumor cells depend upon the formation of angiogenesis, the progress of neovascularization is actually important for cell proliferation, invasion, and metastasis in solid tumors [2]. Currently, a large number of drugs targeting angiogenesis have been approved for the first- and second-line treatment of HCC [3].

Autophagy is a highly regulated catabolic process in cells. Under normal or stress conditions, autophagy participates in the removal of damaged organelles and the transformation of intracellular substances to maintain homeostasis [4, 5]. Dysregulation of autophagy has serious consequences and is related to the development and progression of various diseases, such as infectious neurodegenerative and metabolic diseases, as well as cancer [6]. Moreover, studies have shown that autophagy may be involved in maintaining the occurrence of HCC [7]. In the early stage in the development of HCC, autophagy is against tumor formation by inhibiting inflammation and maintaining genomic stability. Once cancer develops, autophagy may act as a prosurvival mechanism to protect HCC cells from death induced by different types of stimulation, including oxidative stress, and thus maintain cancer progression [8].

miRNAs are short noncoding RNAs of 19-25 nucleotides in length that inhibit target gene expression by specifically binding in a sequence-specific manner to the 3′-UTR regions of the target gene [9]. Abnormal expression of miRNAs is associated with the development of most tumors, such as HCC, and involved in the regulation of tumor cell growth, autophagy, and angiogenesis [10]. miR-559 is one of these miRNAs, which has been demonstrated to be involved in the inhibition of cell proliferation and invasion in HCC cells [11]. PARD3 (zonal defect 3 homolog) is a scaffold protein consisting of an N-terminal structural domain, a C-terminal structural domain, and three PDZ structural domains [12]. PARD3 regulates cell proliferation, migration, and invasion in most cancers, including lung cancer and bladder cancer [13, 14]. Also, activation of PARD3 signaling was associated with the increased autophagy activity and colorectal cancer cell proliferation [15]. It was found that PARD3 was overexpressed in HCC and associated with poor prognosis [16]. However, its role in HCC has not been reported. Bioinformatics analysis revealed that PARD3 is a target gene of miR-559. Thus, in the present study, a series of experiments were performed to investigate the effect of miR-559 and PARD3 on HCC cells, as well as to further clarify the relationship between miR-559 and PARD3.

## 2. Materials and Methods

### 2.1. Cell Lines and Cell Culture

The HCC cell lines (SNU-387, Huh-7, HCCLM3, and MHCC-97H cells) and the normal human liver cell line L02 were provided by ATCC (Manassas, VA, USA). Cells were cultured in high-glucose DMEM (Gibco BRL, USA), with the supplements of fetal bovine serum (10%, FBS) and penicillin/streptomycin (1%; Sigma-Aldrich, St. Louis, MO, USA) at 37°C with 5% CO_2_.

### 2.2. CCK8 Assay

Cell viability was detected by the CCK8 assay. 1∗10^5^ cells were inoculated in 96-well plates for 6 h. Then, cells were transfected with si-RNA or miRNA mimics/inhibitor for 24 h. The medium was removed and replaced with fresh medium containing 10% CCK8 solutions (Meilunbio; China) and cultured for another 1 hour. The optical density at 450 nm was measured with a microplate reader (Biotek, Inc., Woburn, MA, USA).

### 2.3. Cell Transfection

si-RNA for PARD3, miR-559 mimics, or miR-559 inhibitor was obtained from GenePharma (Shanghai, China). Cells were seeded in 6-well plates to 50-60% and transfected for si-RNA or miRNA mimics/inhibitor using RiboFECT™ CP transfection Reagent (Ribobio, Guangzhou, China) as per instructions.

### 2.4. RT-PCR Assay

The TRIzol reagent (Invitrogen, Carlsbad, CA, USA) was used to extract total RNA as per instructions. A Mir-X™ miRNA qRT-PCR TB Green kit (Takara, Japan) and PrimeScript RT reagent kit (Takara) were used for miRNA and total RNA reverse transcription, respectively. Relative level of miR-559 was normalized to U6 expression. RT-PCR was performed using TB Green Premix Ex Taq (Takara) on StepOne Plus Real-Time PCR System (Applied Biosystems, CA, USA). Relative gene levels were normalized to GAPDH expression. The primer sequence is presented in Table 1.

### 2.5. Western Blot Assay

HCC cells were collected and dealt with RIPM lysed buffer to obtain total protein. The protein concentration was measured by using a BCA protein quantification kit (Kerui, Wuhan, China). Then, the proteins were divided by SDS-PAGE and shifted to PVDF (0.45 *μ*m) membranes. Next, the membranes were soaked in nonfat milk (5%). After blocking, the blots were incubated with antibodies against PARD3 (BioVision, Palo Alto, USA; 1 : 1000), LC3-I/II (Cell Signaling Technology (CST), Beverly, MA, USA; 1 : 1000), Beclin1 (Absin, Shanghai, China; 1 : 1000), p62 (Absin, 1 : 1000), VEGF (CST, 1 : 1000), Ang-2 (Santa Cruz, CA, USA; 1 : 1000), and *β*-actin (Absin, 1 : 1000) overnight at 4°C. Finally, bands were incubated with an HRP-conjugated secondary antibody (1 : 2500) (at room temperature, 1 h) and quantitated using bioimaging. The protein level was valued using *β*-actin as a loading control.

### 2.6. HUVEC Tube Formation Assays

In tumor cell-conditioned medium (TCM) preparation, stably transfected Huh-7 cells were cultured in DMEM supplemented with 10% FBS (37°C, 24 h). Then, the medium was changed to DMEM containing FBS (1%) and cultured (48 h). Then, the medium was collected and centrifuged (600 g, 5 min). The detached cells were removed, and the TCM supernatant was then concentrated by using the Millipore 3 kDa Centricon column. The TCM should be used immediately or stored in aliquots at -20°C until use.

In tube formation assays *in vitro*, 5∗10^4^ cells of HUVECs were cultured with TCM for 12 h until tube-like structures formed, at 37°C in 24-well plates coated with Matrigel. Use a light microscope to capture the formation of tubes. Four images were obtained at nonoverlapping locations, and the number of tubes was measured using ImageJ software.

### 2.7. Dual-Luciferase Reporter Assay

Wild-type and mutant luciferase vectors were constructed according to the predicted binding sites of PARD3 and miR-559. Then, cells were cotransfected with miR-559 mimics or NC mimics for 24 h. After transfection, cells were collected and lysed and centrifuged (10,000 g, 5 min). The upper supernatant was collected, and the luciferase activity was detected using the Dual-Luciferase Reporter Assay System (Promega Corporation, Madison, WI, USA).

### 2.8. Target Prediction

The prediction of miRNAs binding with PARD3 was performed using the TargetScan database (http://www.targetscan.org/vert_72/).

### 2.9. Statistical Analysis

Data was analyzed statistically using GraphPad 8.0 and expressed as mean ± SD. The differences between two groups were performed by two-tailed Student's *t*-test or one-way ANOVA followed by post hoc Dunnett's test. *p* < 0.05 was considered statistically significant.

## 3. Results

### 3.1. miR-559 Was Downregulated and PARD3 Was Upregulated in HCC Cells

**Figure 1:**
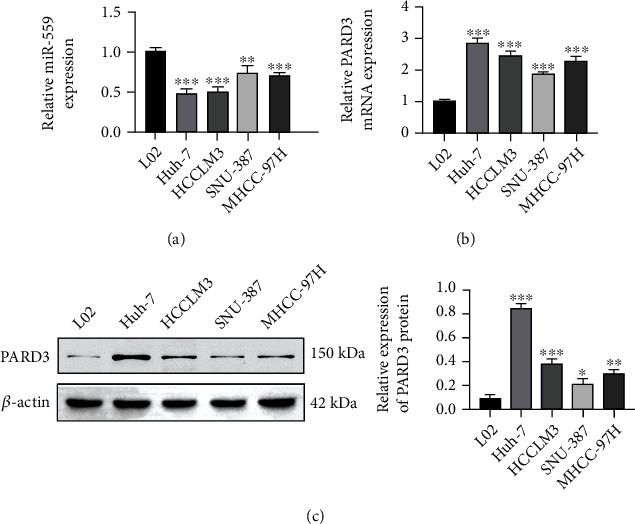
miR-559 was downregulated, and PARD3 was upregulated in HCC cells. (a) The expression level of miR-559 in HCC cells. (b) The mRNA expression of PARD3 in HCC cells. (c) The protein level of PARD3 in HCC cells. The values were obtained from three independent experiments and shown as mean ± SD. ^∗^*p* < 0.05, ^∗∗^*p* < 0.01, and ^∗∗∗^*p* < 0.001, compared with L02 cells.

The expression levels of miR-559 and PARD3 were detected in HCC cell lines (Huh-7, HCCLM3, SNU-387, and MHCC-97H) and the normal liver cell line (L02) by the RT-PCR assay. As shown in Figures 1(a) and 1(b), miR-559 was markedly downregulated, while PARD3 was obviously upregulated in HCC cells. Analysis on protein expression showed consistent results (Figure 1(c)). The Huh-7 cell line showed the lowest expression of miR-559 and the highest expression of PARD3, which was used in the following study to explore the role of PARD3 in HCC and its interaction between miR-559.

### 3.2. miR-559 Overexpression or PARD3 Silencing Inhibited the Proliferation and Autophagy of Huh-7 Cells

**Figure 2:**
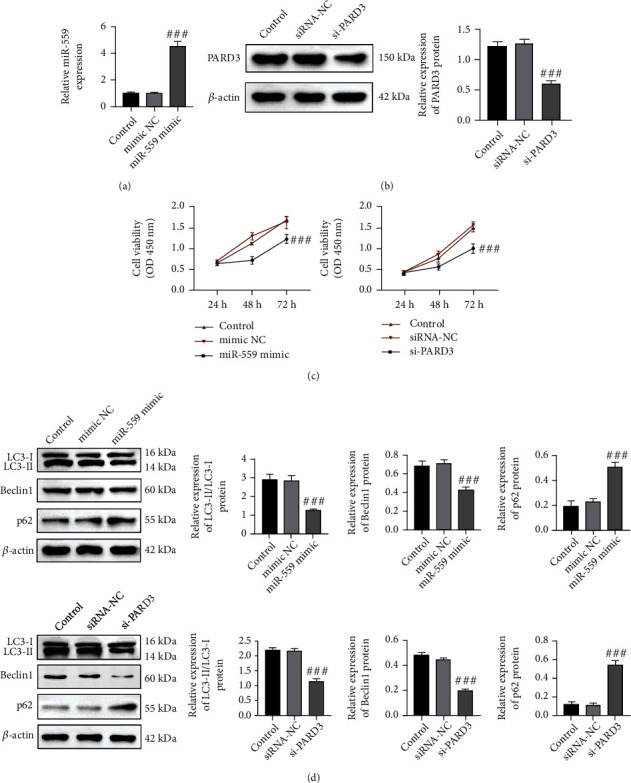
miR-559 overexpression or PARD3 silencing inhibited the proliferation and autophagy of Huh-7 cells. Huh-7 cells were transfected with miR-559 NC/mimics or si-RNA NC/PARD3 for 24 h. (a) The relative expression of miR-559. (b) The relative protein expression of PARD3. (c) Cell viability was detected by the CCK8 assay. (d) The relative protein levels of LC3-II/I, Beclin1, and p62. The values were obtained from three independent experiments and shown as mean ± SD. ^###^*p* < 0.001, compared with mimic NC or siRNA-NC.

To further investigate the effects of miR-559 and PARD3 in Huh-7 cells, Huh-7 cells were transfected with miR-559 mimics or si-PARD3, respectively. The results are presented in Figures 2(a) and 2(b); the mRNA expression of miR-559 and protein expression of PARD3 were, respectively, increased and decreased in Huh-7 cells after being transfected with miR-559 mimics and si-PARD3, validating the successful transfection. Then, the cell viability was measured by the CCK8 assay. Overexpression of miR-559 significantly reduced cell viability, and the similar investigation was found in cells with si-PARD3 (Figure 2(c)). PARD3 overexpression enhanced autophagy and promoted the development of colorectal cancer [15]. The level of LC3-II/I is known as an autophagosomal marker [17]. It has been reported that autophagic overexpression of LC3-II contributes to malignant progression and predicts poor prognosis in HCC [18]. Beclin1 is a key regulator of autophagy, and Beclin1-dependent autophagy has been shown to be associated with the development of HCC [17]. As an important autophagy receptor, P62 participates in the autophagy process. When autophagy activity is weakened or the autophagy system is damaged, P62 protein accumulates in the cytoplasm, and therefore, P62 is considered to be one of the important marker proteins reflecting autophagy activity [19]. In this study, upregulation of miR-559 decreased the expression of LC3-II/I and Beclin1 and increased the expression of p62. PARD3 silencing showed the similar results (Figure 2(d)). These results suggested that miR-559 overexpression or PARD3 downregulation might inhibit the proliferation and autophagy in Huh-7 cells.

### 3.3. Overexpression of miR-559 or Inhibition of PARD3 Reduced the Angiogenesis of Huh-7 Cells

**Figure 3:**
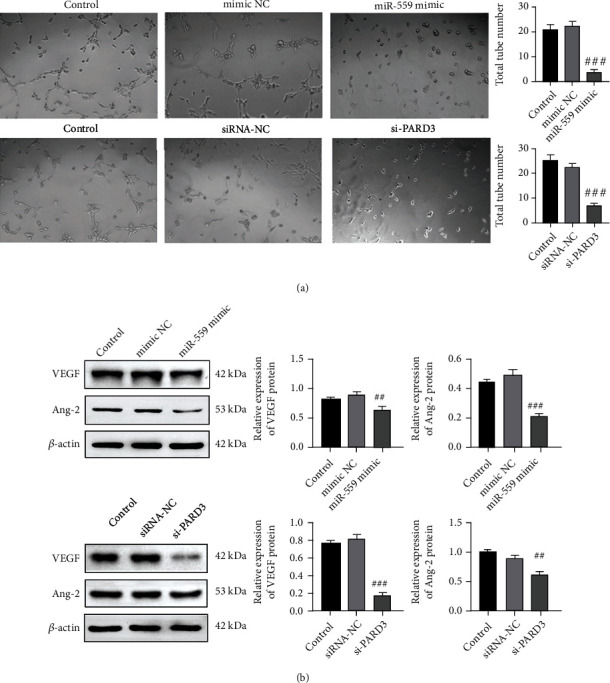
Overexpression of miR-559 or inhibition of PARD3 reduced the angiogenesis of Huh-7 cells. Huh-7 cells were transfected with miR-559 NC/mimics or si-RNA NC/PARD3 for 24 h. (a) *In vitro* HUVEC tube formation assay using conditioned medium and the quantification of tube length in each group. (b) Western blot analysis on the protein expressions of VEGF and Ang-2. The values were obtained from three independent experiments and shown as mean ± SD. ^###^*p* < 0.001, compared with mimic NC or siRNA-NC.

Increasing studies have shown that abnormal angiogenesis is a key process in the development of cancer [20]. Thus, the role of miR-559 or PARD3 was evaluated in the angiogenesis in Huh-7 cells by using the *in vitro* tube formation assay. As shown in Figure 3, HUVECs cultured with TCM from miR-559 mimics or si-PARD3-treated cells developed less capacity for angiogenesis. Studies reported that proangiogenic molecules including VEGF and Ang-2 were involved in cancer development [21]. The results showed that the protein levels of VEGF and Ang-2 were also markedly reduced in HUVECs cultured with TCM treated with miR-559 mimics and si-PARD3. Collectively, these above results suggest that overexpression of miR-559 or downregulation of PARD3 may inhibit the progression of HCC.

### 3.4. PARD3 Was a Target Gene of miR-559

**Figure 4:**
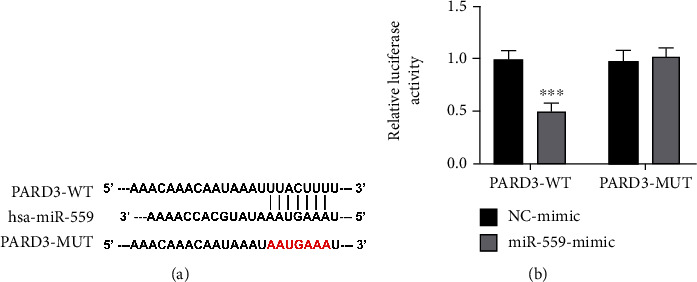
PARD3 was a target gene of miR-559. (a) Target prediction between PARD3 and miR-559. (b) Luciferase reporter constructs containing wild-type or mutated miR-559 paring sites of PARD3 were cotransfected with the miR-559 mimic or mimic NC into Huh-7 cells. The values were obtained from three independent experiments and shown as mean ± SD. ^∗∗∗^*p* < 0.001, compared with mimic NC.

To further explore the correlation between miR-559 and PARD3, TargetScan software was used to analyze the interaction between miRNAs and PARD3. Seven conserved binding sites for miR-559 were identified in the 3′-UTR region of the PARD3 gene. To elucidate whether PARD3 was a direct target of miR-559, a luciferase reporter plasmid containing the 3′-UTR of PARD3 was established. As shown in Figure 4, the luciferase activity was similar in cells cotransfected with miR-559 mimics or negative control mimics in the mutant-type 3′-UTR group. However, the relative luciferase activity with wild-type 3′-UTR was markedly reduced by miR-559 mimics. These results indicated that PARD3 was directly regulated by miR-559.

### 3.5. PARD3 Silencing Reversed the Effect of the miR-559 Inhibitor on Huh-7 Cells

**Figure 5:**
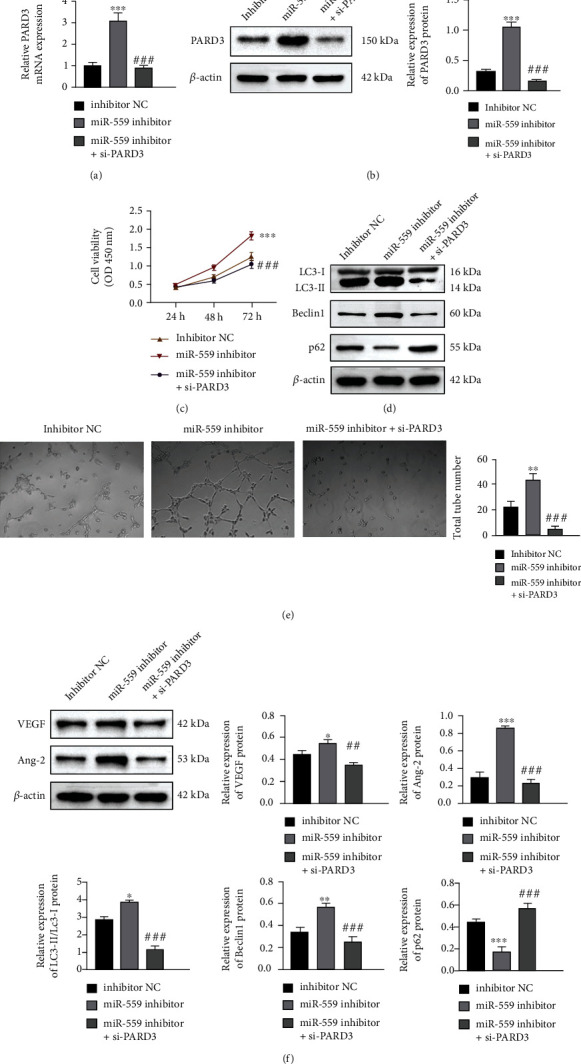
PARD3 silencing reversed the effects of the miR-559 inhibitor on Huh-7 cells. Huh-7 cells were cotransfected with the miR-559 inhibitor and si-PARD3 for 24 h. The mRNA (a) and protein (b) levels of PARD3 in Huh-7 cells. (c) Using the CCK8 assay to detect cell viability. (d) The expression levels of LC3-II/I, Beclin1, and p62 were measured by the western blot assay. (e) HUVEC tube formation assay was proposed using conditioned medium *in vitro*. (f) The protein levels of VEGF and Ang-2 were detected by western blotting in Huh-7 cells. The values were obtained from three independent experiments and shown as mean ± SD. ^∗^*p* < 0.05, ^∗∗^*p* < 0.01, and ^∗∗∗^*p* < 0.001, compared with inhibitor NC; ^##^*p* < 0.001 and ^###^*p* < 0.001, compared with the miR-559 inhibitor.

To further validate whether the effects of miR-559 on Huh-7 cells are related to the direct regulation of PARD3, Huh-7 cells were treated with si-PARD3 and miR-559 inhibitor, and the protein level of PARD3, cell viability, autophagy, and angiogenesis were, respectively, evaluated. Inhibition of miR-559 obviously induced the mRNA and protein expression of PARD3, while si-PARD3 totally blocked this phenomenon (Figures 5(a) and 5(b)). In addition, inhibition of miR-559 enhanced cell proliferation, autophagy, and angiogenesis, but the inhibitory effect of miR-559 on Huh-7 cells was reversed by PARD3 silencing (Figures 5(c)–5(f)). These results suggested that miR-559 regulates cell proliferation, autophagy, and angiogenesis by negatively regulating PARD3.

## 4. Discussion

HCC is a common type of liver cancers, comprising more than 90% of all primary malignant liver cancers [3]. Although HCC has been extensively studied, the underlying mechanisms about its tumorigenesis and progression are still poorly understood due to its complexity. In recent years, accumulating evidence showed that most miRNAs are closely related to the occurrence, metastasis, and poor prognosis of HCC [22]. Moreover, advancements in exploring the molecular mechanism of miRNAs hold the promise of these molecules as attractive targets for the diagnosis and management of HCC [23]. Our study found that miR-559 was downregulated in various HCC cells, while PARD3 was upregulated in HCC cells (Figure 1). In addition, overexpression of miR-559 or knockdown of PARD3 decreased the proliferation of HCC cells (Figure 2(c)). These results suggested that a relationship between miR-559 and PARD3 might be involved in the development of HCC cells.

Autophagy is thought to act as a tumor suppressor to maintain the stability of the intracellular environment. However, after tumorigenesis, autophagy enables cancer cells to survive in the tumor microenvironment and promote tumor growth and development [6, 24]. Therefore, blocking autophagy may be an ideal target for HCC treatment. Our results demonstrated that upregulation of miR-559 or PARD3 silencing inhibited autophagy to restrain HCC growth. The expression of autophagy activation-related proteins was significantly decreased, such as LC3-II/I and Beclin1 [17]. In contrast, the expression of autophagy inhibition-related proteins was significantly increased, such as p62 [25] (Figure 2(d)).

In HCC, the angiogenic pathway is dysregulated, suggesting the involvement of angiogenesis in the development and pathogenesis of HCC [2]. Many vascular growth factors can promote tumor angiogenesis, and the tumor itself can induce the secretion of these vascular growth factors through a variety of ways [26, 27]. In particular, vascular endothelial growth factor (VEGF) mainly regulates the growth and survival of endothelial cells, which promotes tumor angiogenesis and accelerates tumor development since it induces dormant tumor cells to rejuvenate the cell cycle process [28]. This study showed that overexpression of miR-559 or PARD3 knockdown inhibited tumor angiogenesis by decreasing the expression of VEGF and Ang-2 (Figure 3), two representative angiogenic factors.

Since the main function of miRNAs is to inhibit the expression of target genes by interfering with their transcription via binding to the 3′-UTR region of target genes [29]. In combination with bioinformatics prediction and dual luciferin reporting experiments, PARD3 was identified as a target gene for miR-559 (Figure 4). Therefore, the high expression of PARD3 in HCC cells was related to the downregulation of miR-559. Meanwhile, it is demonstrated that miR-559 inhibition induced the protein level of PARD3, while cell viability, autophagy, and angiogenesis were promoted in HCC cells by miR-559 inhibition. However, knockout of PARD3 reversed these phenomena. Thus, the role of miR-559 on cell viability, autophagy, and angiogenesis might be through the regulation of PARD3 expression.

In conclusion, all these results suggested that PARD3 is a target gene of miR-559. Overexpression of miR-559 inhibits the proliferation, autophagy, and angiogenesis in HCC cells via reducing the expression of PARD3, thus contributing to the development of HCC resistance. Therefore, miR-559 can serve as a novel therapeutic target for HCC.

## Figures and Tables

**Table 1:** The information of primers.

Gene name	Forward (5′-3′)	Reverse (3′-5′)
*miR-559*	CCTGGGACCCCATTATCCTT	TGCTGTCCACAGTGTGTTTG
*Pard3*	CAGACAGAACTACTAACTTCGCC	ATGCCTCGGATGAAGAGTCCT
*U6*	CTCGCTTCGGCAGCACA	AACGCTTCACGAATTTGCGT
*Gapdh*	TCAAGATCATCAGCAATGCC	CGATACCAAAGTTGTCATGGA

## Data Availability

All data generated or analyzed during this study are included in this published article.
